# Self-Optimized Biological Channels in Facilitating the Transmembrane Movement of Charged Molecules

**DOI:** 10.1155/2016/1657679

**Published:** 2016-02-28

**Authors:** V. T. N. Huyen, Le Bin Ho, Vu Cong Lap, V. Lien Nguyen

**Affiliations:** ^1^Institute for Bio-Medical Physics, 109A Pasteur, 1st District, Ho Chi Minh City 710115, Vietnam; ^2^VAST/Institute of Physics, 1 Mac Dinh Chi, 1st District, Ho Chi Minh City 710116, Vietnam

## Abstract

We consider an anisotropically two-dimensional diffusion of a charged molecule (particle) through a large biological channel under an external voltage. The channel is modeled as a cylinder of three structure parameters: radius, length, and surface density of negative charges located at the channel interior-lining. These charges induce inside the channel a potential that plays a key role in controlling the particle current through the channel. It was shown that to facilitate the transmembrane particle movement the channel should be reasonably self-optimized so that its potential coincides with the resonant one, resulting in a large particle current across the channel. Observed facilitation appears to be an intrinsic property of biological channels, regardless of the external voltage or the particle concentration gradient. This facilitation is very selective in the sense that a channel of definite structure parameters can facilitate the transmembrane movement of only particles of proper valence at corresponding temperatures. Calculations also show that the modeled channel is nonohmic with the ion conductance which exhibits a resonance at the same channel potential as that identified in the current.

## 1. Introduction

Biological channels are responsible for regulating the fluxes of ions and molecules (hereafter referred to as particles for short) across membranes and, therefore, are critically important for the cell functioning [1]. As well-known, these protein channels are very efficient in the sense that they support a very fast, selective, and robust across membrane transport, regardless of environment fluctuations [2]. Surprisingly, such privileged properties have been observed even in the case of large water-filled channels, where the particle transport does not involve the use of metabolic energy or conformational changes and was assumed to be simply diffusive [3]. Understanding the nature of this channel-facilitated particle movement (CFPM) is crucially important from the fundamental molecular biology as well as the application point of view (many modern drugs are developed in the way of using the ion-channels to enhance their efficiency; see, e.g., [4–6]).

Experimentally, there are accumulative data showing that the observed CFPM really resulted from some interaction between the moving particle and the channel interior-lining [7, 8]. Recent advancements of high-resolution current recording enable single-channel measurements that provide directly a living picture of how an individual channel functions and, therefore, shed light on the characteristics of channel current in dependence on different (channel and environment) parameters [7–9]. However, revealing exactly the nature of channel-particle interaction as well as the mechanism of CFPM is still very experimentally problematic due to the puzzled complexities related to both the channel structure and the measurement systems.

Theoretically, to describe the CFPM several models have been suggested. Considering the one-dimensional (1D) diffusion model with a position-dependent diffusion coefficient, Berezhkovskii et al. supposedly introduced a square potential well, spanning the whole channel length, that brings about a channel-particle interaction [10–13]. It was then shown that at a given solute concentration difference there exists an optimum potential well depth that can maximize the particle current, facilitating the channel function. In this model (i) the channel is assumed to be large enough so that all the effects related to the particle size can be omitted, (ii) a single-particle diffusion is considered, neglecting all many particle correlations, and, particularly, (iii) no realistic potential was assigned as the source for the square potential well introduced. Bauer and Nadler considered a similar 1D diffusion model with a square potential well that is however associated locally with only the particle bound temporarily inside the channel [14]. Using the macroscopic version of Fick's equation, it was then demonstrated that a transport increase always occurs for any square potential wells. However, as already noted by the authors, the square potential well exploited in this model is also rather crude and a more realistic potential should be found [14]. From the very other point of view, Kolomeisky models the channel as a set of discrete binding sites arranged stochastically [15]. In such the discrete-state model the particles are assumed to hop along the binding sites in translocations across the channel and the optimum current may be achieved depending on the spatial distribution of binding-sites and the site-particle interactions [15, 16]. This model is so simple that the main dynamic properties of the problem can be calculated exactly. It was also demonstrated that the discrete-state model [15] and the continuum diffusion model [10] are closely related and can be effectively mapped into each other [17]. Nevertheless, like the square potential well in the continuum models [10, 14], the nature of the binding sites (a kind of channel-particle interaction) and the hopping mechanism of particles in the discrete-state model [15] still need to be identified.

Importantly, in all the models mentioned [10, 14, 15] the channel-particle interaction (which was expressed by a square potential well or a binding site) is generally viewed as the crucial condition for the transmembrane transport to be facilitated (see also [18]). Note again that in these models the particle motion is merely considered one-dimensional. Recently, Dettmer et al. have measured the diffusivity of spherical particles in closely confining, finite length channels [19]. Measurements demonstrated a strongly anisotropic diffusion in the channel interior: while the diffusion coefficient parallel to the channel axis remained constant throughout the entire channel interior, the perpendicular diffusion coefficient showed an almost linear decrease from the axis towards the channel wall. These observations put forward a need for the two-dimensional (2D) description with direction-dependent diffusion coefficients when studying the movement of particles inside a large channel. Furthermore, experimentally, the single-channel kinetics was extensively studied at different external voltages [20, 21]. And, the experimental sublinear current-voltage (*I*-*V*) characteristics reported in [22, 23] is often used as one of the basic requirements for theoretical models [24].

In the present paper we consider a 2D diffusive movement of particles through a large water-filled channel, taking into account an anisotropy of diffusion coefficients as observed in [19] and an influence of external voltage as discussed in [20, 24]. The channel is modeled as a cylinder characterized by three structure parameters: radius, length, and surface density of negative charges of channel interior-lining. The potential created by this charged interior-lining inside the channel is exactly calculated. It causes the “channel-particle interaction” that plays a key role in facilitating the transmembrane particle movement. Solving the 2D stochastic Langevin equation for the model suggested we systematically analyze the typically dynamical characteristics of particles such as the translocation probabilities, the translocation times, the currents, and the channel ion conductance under the influence of various factors: the channel-induced potential, the external voltage, or the difference in reservoir particle concentrations. It was particularly shown that to facilitate the transmembrane particle movement the channel should be reasonably self-optimized with appropriate structure parameters so that its potential coincides with the resonant one. In addition, this facilitation is very selective in the sense that a channel of definite structure parameters can facilitate the transmembrane movement of only particles of proper valence at corresponding temperatures. So, the model suggests that facilitating the transmembrane particle movement is an intrinsic property of biological channels. This property is independent of the external factors such as the external voltage or the bulk particle concentration gradient, though these factors may strongly influence the magnitude of various particle dynamical characteristics.

The paper is organized as follows.  Section 2 introduces the 2D diffusion model for the problem under study, including the motion equation with an exact expression of the channel-induced potential, and describes the calculating method.  Section 3 presents the main numerical results obtained. These results are discussed in great detail, showing the influence of various factors on the particle dynamical characteristics. A particular attention is given to the self-optimized property of the channels in facilitating the transmembrane particle movement. The paper concludes with a brief summary in Section 4.

## 2. Model and Calculating Method

**Figure 1:**
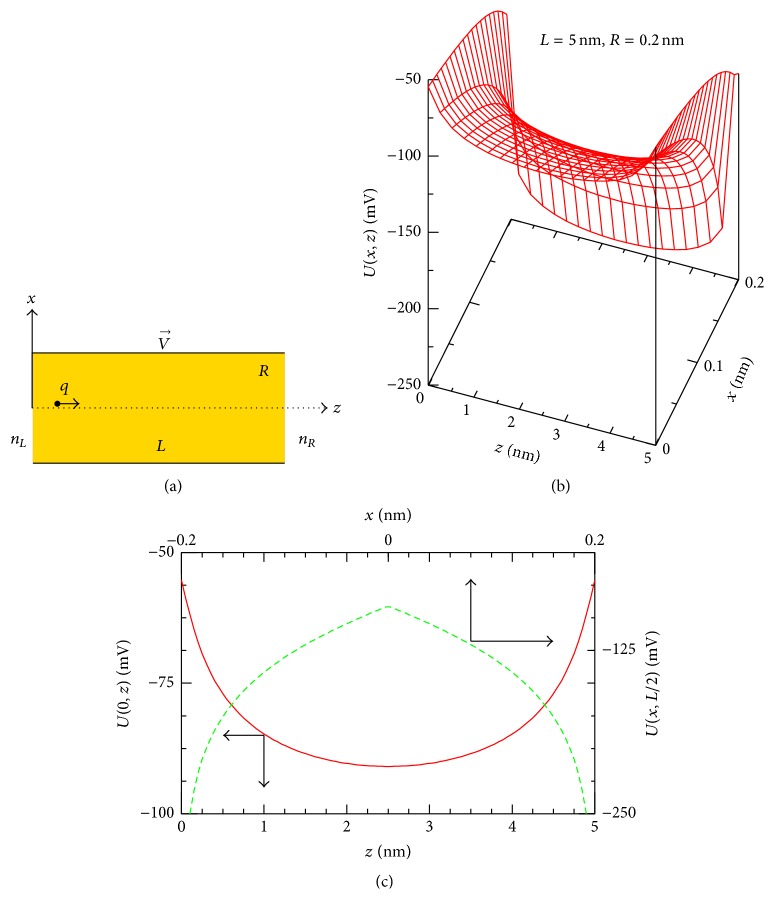
(a) Model of the cylindrical channel under study. (b) Channel-induced potential *U*(*x*, *z*) of (3) is plotted for the channel with *R* = 0.2 nm, *L* = 5 nm, and *σ* = −0.1 C/m^2^ (note: *U*(*x*, *z*) is symmetrical with respect to the sign of *x*); (c) the *U*(0, *z*) potential well (red-solid line, see the left and bottom axes) and the *U*(*x*, *L*/2) potential barrier (green-dashed line, see the right and top axes) for the potential *U*(*x*, *z*) in (b). The potential *U*_0_ ≡ *U*(0, *L*/2)≈−90.6 mV in this case.

We consider a cylindrical channel of length *L* and radius *R* that connects the two reservoirs with particle concentrations *n*_*L*_ and *n*_*R*_ as schematically drawn in Figure 1(a). The channel interior-lining carries negative charges which are for simplicity assumed to be continuously and regularly distributed with a surface density *σ*. (The cation channels are believed to contain a net negative charge in the pore lining region of the protein [25]. In the case of potassium and gramicidin channels this is due to the partially charged carbonyl oxygens [1, 25].) These negative surface charges create an electrostatic potential *U* which affects the movement of particles inside the channel. Particles are assumed to diffuse independently, neglecting any many-particle correlation. In addition, the diffusivity of a particle inside the channel is assumed to be anisotropic with the two different diffusion coefficients, *D*_*z*_ (parallel with) and *D*_*x*_ (perpendicular to the channel axis). Following [19], we assume that (i) *D*_*z*_ is constant throughout the channel cylinder [0 ≤ |*x*| < *R* and 0 ≤ *z* ≤ *L*] and somewhat smaller than the diffusion coefficient *D*_0_ in the bulk, *D*_*z*_ = *αD*_0_ with 0 < *α* < 1 (we choose in the present work *α* = 0.5 for definition) and (ii) *D*_*x*_ linearly decreases as *x* going from the channel axis (where the diffusion is isotropic) to the channel wall, *D*_*x*_ = [1 − (|*x*|/*R*)]*D*_*z*_.

The model also involves a longitudinal voltage *V*, that is, the difference in electrical potential between the two channel ends, that may include the intrinsic membrane potential [1] and/or some externally applied voltage [20]. This voltage drives the particles moving along the channel. For definition, we assumed that the voltage *V* is directed from the left to the right (in Figure 1(a)) and the charge *q* carried by a particle is positive.

Actually, due to the cylindrical symmetry of the channel model suggested, the motion of a particle inside a channel can be effectively described by the 2D stochastic differential equation (Langevin equation for overdamped motion):

(1) γzz00γxxz˙tx˙t=−q∂zUx,z∂xUx,z+qVL0+ξztξxt,

where −*R* < *x*(*t*) < *R* and 0 ≤ *z*(*t*) ≤ *L* are the 2D coordinates of the particle at *t* time, $\dot{x}\equiv dx/dt$, *γ*_*xx*_(*γ*_*zz*_) is the drag coefficient in the *x*(*z*) direction, *U*(*x*, *z*) is the potential created by the charged channel lining, *qV*/*L* is the voltage-induced force acting on a particle of charge *q* in the *z*-direction, and *ξ*_*x*_(*t*)(*ξ*_*z*_(*t*)) is the random force in the *x*(*z*) direction which is as usual assumed to have a zero mean and a white noise correlation:

(2) ξνt=0,ξνtξνt′=2Dνδt−t′,ν=x,z.

It is here worth mentioning the Stokes-Einstein relation between the diffusion coefficient *D*, the drag coefficient *γ*, and the absolute temperature *T* of a medium, *Dγ* = *k*_*B*_ *T*, where *k*_*B*_ is the Boltzmann constant.

In (1) we need to identify the potential *U*(*x*, *z*) inside the channel. Within the model considered, as mentioned above, *U* is the electrostatic potential created by the charged lining of a cylindrical channel. By solving the fundamental electrostatic problem for a charged cylinder of finite sizes, we can exactly derive an analytical expression of  *U* as a function the (*x*, *z*)-coordinates [0 ≤ *x* < *R* and 0 < *z* < *L*]:

(3) Ux,z=Rσπϵ0ϵ1+π2·ln⁡z+x−R2+z2z−L+x−R2+z−L2−ln⁡z+x+R2+z2z−L+x+R2+z−L2,

where *R*, *L*, and *σ* are the channel structure parameters defined above, *ϵ*_0_ is the vacuum permittivity, and *ϵ* is the dielectric constant of the water in the interior of the channel (whereas the dielectric constant of the water in the channel interior is often assumed to be lower than the bulk value, generally, it is still remains an issue due to the lack of experimentally solid data [24]. Ignoring all of these unsolved complications, for definition, we choose for *ϵ* the same value of the dielectric constant of the bulk water, *ϵ* ≈ 80. It is important to note that though the magnitude of the potential *U* in (3) does depend on *ϵ*, the *U*(*x*, *z*) behavior does not, and, therefore, all qualitative conclusions of this work are unaffected by a choice of *ϵ*-value). Note that the potential *U*(*x*, *z*) is symmetrical with respect to the sign of *x*.

As an example, Figure 1(b) shows the potential *U*(*x*, *z*) of (3) for the channel with *R* = 0.2 nm, *L* = 5 nm, and *σ* = −0.1 C/m^2^. At a given *x*-coordinate, *U*(*z*) behaves as a symmetrical potential well with the absolute minimum at *z* = *L*/2. On the contrary, given a *z*-coordinate, the *U*(*x*)-curve describes a symmetrical potential barrier with the absolute maximum at *x* = 0 (see, e.g., *U*(0, *z*) as a function of *z* (bottom and left axes) and *U*(*x*, *L*/2) as a function of *x* (top and right axes) in Figure 1(c)). While the well shape of the channel potential *U*(*x*, *z*) in the *z*-direction directly affects the movement of particles across the channel (as will be seen below), its barrier shape in the *x*-direction demonstrates a noticeable role of the transverse motion in the anisotropic 2D diffusion model considered.

As a consequence of the observed symmetrical shape, the potential *U*(*x*, *z*) can be characterized by its value at the center of the channel, (*x* = 0, *z* = *L*/2), where

(4) $$\begin{array}{c} U\left(\;0,\frac{L}{2}\;\right)=\frac{R\sigma }{2\epsilon_{0}\epsilon }\ln\left[\;\frac{\sqrt{4R^{2}+L^{2}}+L}{\sqrt{4R^{2}+L^{2}}-L}\;\right]\equiv U_{0}. \end{array}$$

This potential value *U*_0_ is uniquely determined by the channel structure parameters (*L*, *R*, and *σ*) and can be used to characterize the potential *U*(*x*, *z*) on the whole: each channel creates unique *U*(*x*, *z*) and each *U*(*x*, *z*) has a unique *U*_0_. As an intrinsic characteristic of the channel, the quantity *U*_0_ will be used below as a typical measure of the channel potential *U*(*x*, *z*). Figure 1(c) indicates the potential *U*_0_ ≈ −90.6 mV for the channel potential *U*(*x*, *z*) examined in this figure.

Thus, as an extension of the model suggested by Berezhkovskii et al. [10–13], the present model is distinguished by the main factors as follows: (i) the diffusion is anisotropically two-dimensional (see (1)), (ii) the negatively charged channel interior-lining creates inside the channel a potential that leads to the first term in the right hand of (1) and that can be exactly identified as a function of only channel structure parameters (see (3)), and (iii) the external voltage causes a driving force expressed by the second term in the right hand of (1). Further, the study will be focused on showing how these factors affect the dynamical characteristics of particles moving through the channel. The dynamical characteristics we are here interested in include the translocation probabilities, the translocation times, the particle current, and the ion conductance. To calculate these quantities we have to solve (1). Reasonably, this stochastic equation can be solved numerically by using the molecular dynamics method [26].

A particle enters the channel from either the left (*z* = 0) or the right (*z* = *L*) at random with the probabilities proportional to the reservoir particle concentration *n*_*L*_ or *n*_*R*_, respectively (Figure 1(a)). The initial *x*-coordinate (−*R* < *x*(*t* = 0) < *R*) and the initial velocity components ($\dot{z}(0)$ and $\dot{x}(0)$) are randomly given, following the standard molecular dynamics simulation procedure [26]. Started from the given initial conditions, a discrete trajectory of the particle is step by step constructed. Given the channel potential *U*(*x*, *z*) and the external voltage *V*, in each time step (Δ*t*) the random forces, *ξ*_*x*(*z*)_, are independently generated and then the final coordinates and velocity of the particle are determined from (1) using the well-known Euler scheme [27, 28]. In the *x*-direction the full reflection condition is applied every time when a particle runs into the channel wall, *x* = ±*R*. In the other direction, once the *z*-coordinate is out of the range [0, *L*], the data for the simulated particle is fixed and this particle is no longer followed. The next particle enters the channel and undergoes a diffusion process in the same way as described above. The number of particles involved in getting each of the average values of studied dynamical quantities is so large that for all the data points presented below the error bar nowhere exceeds the symbol size [≈10^5^ to 10^7^ particles depending on the quantity and the direction of movement investigated]. The time step is taken to be Δ*t* = 0.0005*τ*_0_, which is believed to be small enough. The dynamical quantities we are interested in, as mentioned above, include the translocation probabilities, the average translocation times, the net particle current, and the ion conductance.

Actually, the calculating method we exploit in this study is the Brownian dynamics. By solving the Langevin equation, this method is rather appropriate for the problem of interest. A systematical classification of computational approaches proposed and employed for studies of ion-channels can be found in the review paper [29]. Here, in solving numerically (1), for convenience we choose *L* as the unit of length, *τ*_0_ = *L* ^2^/*D*_0_ as the unit of time, and *k*_*B*_ *T* as the unit of energy. So, for example, if *L* = 5 nm and *D*_0_ = 3 · 10^−10^ m^2^/s [17], then *τ*_0_ ≈ 8.3 · 10^−8^ s. We recall that *k*_*B*_ *T* ≈ 8.617 · 10^−5^ eV for *T* = 1°K.

## 3. Numerical Results and Discussions

In presenting simulation results we introduce for short the symbols *u*_0_ ≡ *qU*_0_/*k*_*B*_ *T* (referred to as the effective channel potential) and *v* ≡ *qV*/*k*_*B*_ *T* (referred to as the effective external voltage). So, the defined parameters *u*_0_ and *v* also contain the particle charge *q* and the medium temperature *T*. We should keep this in mind when discussing the role of the channel potential in facilitating the transmembrane particle movement. Additionally, for definition, in all the figures relating to the translocation probabilities and the average translocation times the parameters *R* and *n*_*L*(*R*)_ are kept constant: *R* = 0.04 (in unit of *L*) and *n*_*L*(*R*)_ = 145(15) mM [1]. Influences of these parameters will be later discussed when analyzing the net current (Figure 6).

Let us first examine obtained results for the translocation probabilities which are separately calculated for the particles moving through the channel from the left to the right (*P*_*L*_) and for those moving in the opposite direction (*P*_*R*_) (see Figure 1 with the *V*-direction indicated). In simulations, the probability *P*_*L*_ (or *P*_*R*_) is determined as the ratio of the number of particles that passed through the channel to the total number of particles that entered the channel from the left (or right).

**Figure 2:**
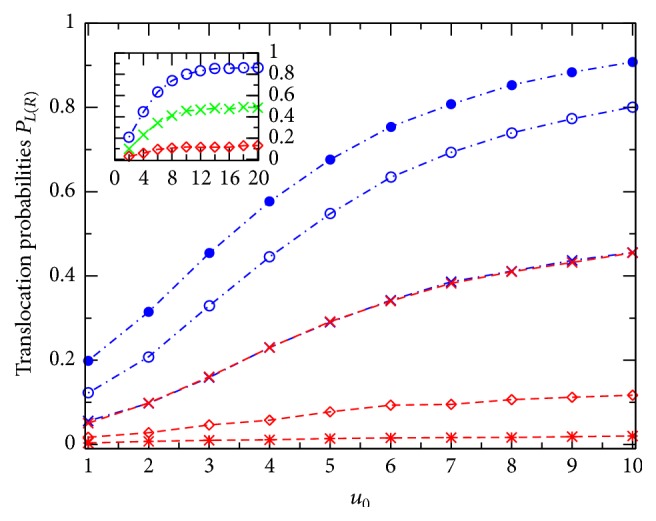
Translocation probabilities *P*_*L*_ (blue dash-dotted lines) and *P*_*R*_ (red dashed lines) are plotted against *u*_0_ ≡ *qU*_0_/*k*_*B*_ *T* for the channels of the same *R*, *n*_*L*_, and *n*_*R*_, but at different voltages *v* ≡ *qV*/*k*_*B*_ *T*: 0(×), 2(∘ and ⋄), and 4(• and *∗*). The points are the simulation results, whereas the lines are drawn as a guide for the eyes. Inset: *P*_*L*(*R*)_ becomes saturated at larger *u*_0_ [*R* = 0.2 nm, *L* = 5 nm, *n*_*L*_ = 145 mM, *n*_*R*_ = 15 mM].

Figure 2 shows *P*_*L*_ (blue dash-dotted lines) and *P*_*R*_ (red dashed lines) plotted against *u*_0_ for the channels at different effective voltages *v*: 0(×), 2(∘ and ⋄), and 4(• and *∗*). Generally, this figure demonstrates that with increasing *u*_0_ both the translocation probabilities, *P*_*L*_ and *P*_*R*_, increase steadily first (see main figure) and then become saturated (see the inset). Such the *P*_*L*(*R*)_-versus-*u*_0_ behavior is observed at any voltage *v*. In the case of zero *v*, due to the left-right symmetry of the potential *U*(*x*, *z*) of (3) the two curves, *P*_*L*_ and *P*_*R*_, are totally coincidental and the common curve may be in a qualitative comparison with Figure  3 in [10] (where the considered diffusion is one-dimensional and the potential well is square). Note that with the chosen direction of *V* (Figure 1; *q* is positive) the external voltage raises *P*_*L*_ (two higher curves) while suppressing *P*_*R*_ (two lower curves), compared to the case of *v* = 0 (the middle curve).

**Figure 3:**
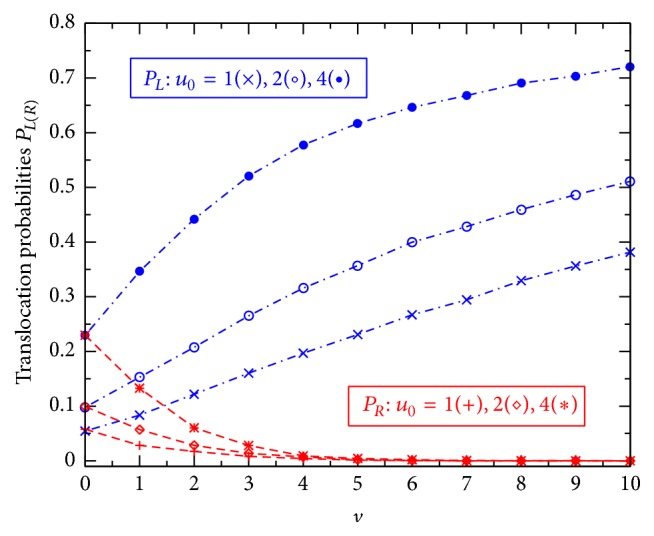
Translocation probabilities *P*_*L*_ (blue dash-dotted lines) and *P*_*R*_ (red dashed lines) are plotted against *v* for the channels with different effective potentials *u*_0_: 1(× and +), 2(∘ and ⋄), and 4(• and *∗*). Other parameters and symbols are the same as in Figure 2.

The external voltage effects can more clearly be seen in Figure 3 where the probabilities *P*_*L*(*R*)_ are presented as the functions of *v* for the channels with different *u*_0_: 1(× and +); 2(∘ and ⋄); and 4(• and *∗*). At zero *v* the two probabilities *P*_*L*(*R*)_ associated to the same *u*_0_ are of equal value (two corresponding curves are started from the same point). With increasing *v* the probability *P*_*L*_ smoothly rises, while the probability *P*_*R*_ strongly descends. At *v* ≥ 5 the probabilities *P*_*R*_ become practically vanished for all the channels under study (no particle can move through the channel in the right-to-left direction). The probability *P*_*L*_, on the contrary, continues to grow with the tempo that gradually slows down at higher *v*. Calculations reveal that even at *v* = 100 the channels are still not perfectly transparent for the positively charged particles moving along the external voltage direction [*P*_*L*_ = 0.98 or 0.95 for *u*_0_ = 4 or 1, resp.].

Next, we consider another fundamental characteristic, the average translocation time. In accordance with the probabilities *P*_*L*(*R*)_ studied in Figures 2 and 3, we separately calculated the average translocation times for the particles moving through the channel from the left to the right (*τ*_*L*_) and for those moving in the opposite direction (*τ*_*R*_). In simulations, we count the time each of the simulated particles spends inside the channel. The average translocation time *τ*_*L*_ (or *τ*_*R*_) is then obtained by averaging these spending times over all the particles that passed through the channel from the left to the right (or from the right to the left).

**Figure 4:**
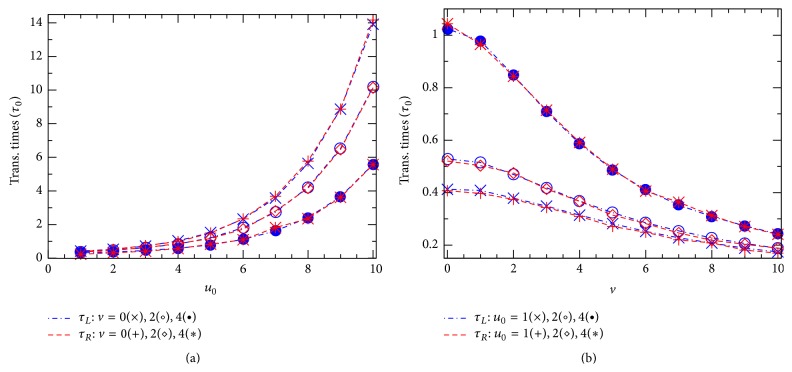
Translocation times *τ*_*L*_ (blue dash-dotted lines) and *τ*_*R*_ (red dashed lines) are plotted against *u*_0_ at different *v*: 0(× and +), 2(∘ and ⋄), and 4(• and *∗*)  (a) and *τ*_*L*(*R*)_ versus *v* at different *u*_0_: 1(× and +), 2(∘ and ⋄), and 4(• and *∗*) (b). Other parameters are the same as in Figure 2.

Figure 4 shows how obtained translocation times *τ*_*L*(*R*)_ vary with the effective potential *u*_0_ (Figure 4(a)) or the effective voltage *v* (Figure 4(b)). Interestingly, in all the cases studied in both the figures, Figures 4(a) and 4(b), the two points, corresponding to *τ*_*L*_ and *τ*_*R*_, are practically coincided. So, our 2D simulations suggest a general equality, *τ*_*L*_(*u*_0_, *v*) = *τ*_*R*_(*u*_0_, *v*), that should be always valid in the model studied regardless of the shape of the channel potential *U*(*x*, *z*) as well as the presence of the external voltage *V*. This really causes some surprise, noting the directed influence of the voltage *V*. Actually, a similar equality of the two average translocation times has been previously suggested in [11], but it was there relating to the 1D diffusion model without any external voltage. Figure 4 thus allows us to deal with the two times *τ*_*L*_ and *τ*_*R*_ as a single average translocation time that will be below denoted simply by *τ*.

The fact that the channel potential *u*_0_ raises the translocation time *τ* in Figure 4(a), while also raising the translocation probabilities in Figure 2, might cause some surprise. Actually, as will be seen below, it turns out that a competition between these two seemingly contrary effects of the potential *u*_0_ leads to the most important phenomenon in the ion-channel physics, the CFPM. Comparing the points from three curves with different voltages *v*, we learn that in the region of large *u*_0_ (*u*_0_ ≥ 6 in Figure 4(a)) the time *τ* decreases almost linearly as *v* increases from 0 to 4. In a wider range of *v*, Figure 4(b) shows that the larger the effective potential *u*_0_, the stronger the relative effect of *v* on *τ*. In the limit of high external voltage when the *v*-induced driving force becomes to dominate all other terms in the right hand in (1), the translocation time should depend on *v* as $\tau \propto 1/\sqrt{v}$.

While the question of the particular time that most relevantly describes the transmembrane transport and that can be directly measured is still under discussion [14], the net current has always served as the most important quantity that should be determined theoretically in close comparison with experimental measurements. For the problem under study, the net particle current is determined as the average number of particles the two reservoirs actually exchanged via the channel in a unit of time (*τ*_0_).

**Figure 5:**
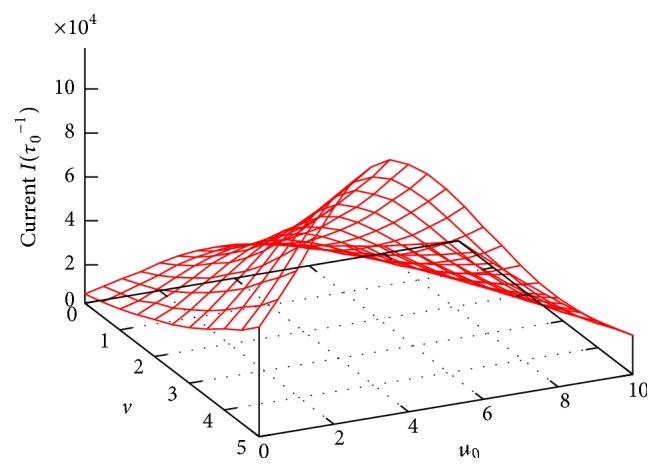
3D plot of the current *I* as a function of *u*_0_ and *v*. Note on the resonant behavior of the *I* versus *u*_0_ curves at different voltages *v*. Other parameters are the same as in Figure 2.

Figure 5 presents a 3D plot of the current *I* in dependence on the effective channel potential *u*_0_ and the effective external voltage *v*. Remarkably, contrary to the monotonic behaviors of *P*_*L*(*R*)_ and *τ* in Figures 2–4, Figure 5 shows clearly a resonant behavior of the current *I*: for a given voltage *v* in the *I* versus *u*_0_ curve there always has an impressively absolute maximum at some resonant channel potential, *u*_0_ = *u*_*m*_. We recall that *u*_0_ ≡ *qU*_0_/*k*_*B*_ *T* with *U*_0_ uniquely determined by the channel structure parameters (*L*, *R*, and *σ*). So, the maximum observed in Figure 5 implies that for given *q* and *T* to successfully facilitate the transmembrane particle movement the channel has to be optimized with the appropriate structure parameters so that its potential *U*(*x*, *z*) coincides with the resonant one. For example, for *q* = 1 and *T* = 300 K, to own the resonant potential of *u*_*m*_ = 3.5 as seen in Figure 5, the channel should be self-optimized with the following structure parameters: *L* = 5 nm, *R* = 0.2 nm, and *σ* = −0.1 C/m^2^ (given *ϵ* = 80 as stated above).

**Figure 6:**
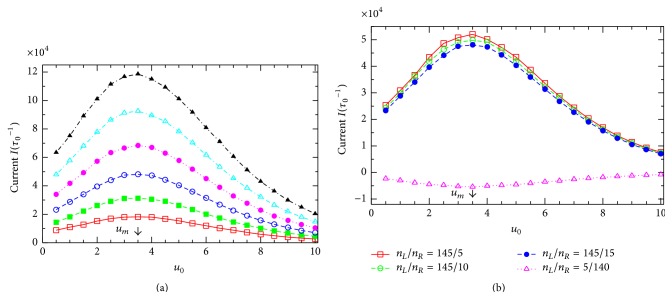
Resonant channel potential *u*_0_ = *u*_*m*_ is an intrinsic characteristic of the channel. (a) *I* versus *u*_0_ curves extracted from Figure 5 for some values of *v* (from bottom: *v* = 0,1, 2,3, 4, and 5). All these curves show their maximum at the same resonant potential, *u*_0_ = *u*_*m*_ (indicated by the arrow). (b) *I* as a function of *u*_0_ at *v* = 2 for various values of the ratio *n*_*L*_/*n*_*R*_ (from top: *n*_*L*_/*n*_*R*_ = 145/5, 145/10, 145/15, and 5/140). The resonant potential *u*_0_ = *u*_*m*_ (indicated by the arrow) is independent of reservoirs particle concentration ratio and coincides with *u*_*m*_ determined in Figure 6(a). Note: in the case of *n*_*L*_/*n*_*R*_ = 5/140 the current is negative (flowing from right to left in Figure 1(a)) and reaches the largest magnitude at the same *u*_0_ = *u*_*m*_.

To see whether the resonant potential *u*_*m*_ depends on the external voltage *v*, we depict in Figure 6(a) some *I*(*u*_0_)-curves extracted from Figure 5 at various *v*. Surprisingly, the resonant channel potential *u*_*m*_ (indicated by the arrow) is practically the same for all the curves at different voltages *v*. Actually, the fact that *u*_*m*_ is independent of *v* can be seen right in Figure 5 for all the values of *v* under study. Further, we check if the resonant potential *u*_*m*_ depends on another important external parameter, the difference in particle concentration between the two reservoirs.  Figure 6(b) presents the *I*(*u*_0_)-dependence for several values of the ratio *n*_*L*_/*n*_*R*_. In the cases of *n*_*L*_/*n*_*R*_ = 145/5, 145/10, and 145/15 (taken from Table  12.1 in [1]), all the particle concentration gradients are directed along the external voltage *V*, that is, from the left to the right in Figure 1(a), and, therefore, the currents are always positive (see the higher three curves). On the contrary, in the case of the lowest curve in Figure 6(b) for *n*_*L*_/*n*_*R*_ = 5/140 (e.g., for *K* ^+^-channels [1]), the particle concentration gradient is directed from the right to the left in Figure 1(a) and, consequently, the current becomes negative (note that in this case the concentration gradient is strong while the external voltage is relatively small, *v* = 2). Importantly, all the curves for various *n*_*L*_/*n*_*R*_ in Figure 6(b) show the maximums in magnitude at the same value of *u*_0_ that exactly coincides with the resonant potential *u*_*m*_ determined in Figure 6(a). Thus, we arrive at an important remark: at given *q* and *T*, the resonant potential is entirely determined by the channel structure parameters. It is an intrinsic property of the channels and can not be affected by the external factors such as the external voltage or the particle concentration gradient.

Thus, in the present model, CFPM appears to be a self-optimized property of biological channels: to facilitate the transmembrane particle movement, the channels should be intrinsically optimized with appropriate structure parameters. Additionally, facilitating the transmembrane transport is very selective in the sense that a channel of definite structure parameters can facilitate the transmembrane transport of only particles of proper valence at corresponding temperatures.

**Figure 7:**
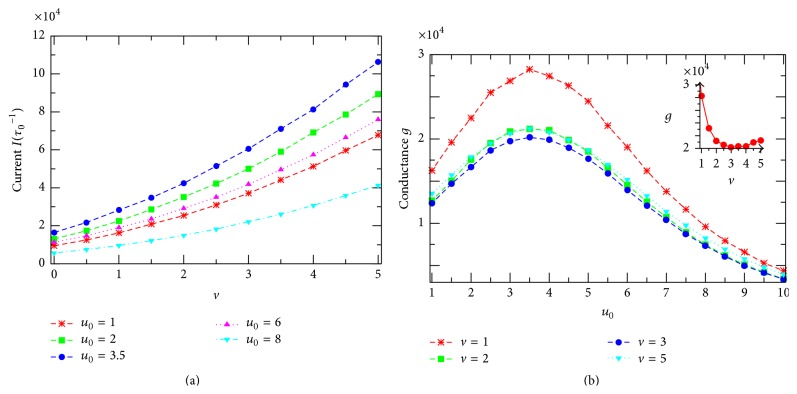
(a) The current *I* is plotted against the voltage *v* [*I*-*V* characteristics] for channels with different potentials *u*_0_ (indicated in the figure). All *I*-*V* curves show a sublinear behavior. (b) The channel ion conductance *g* as a function of the channel potential *u*_0_ at various voltages *v* (given in the figure). At any *v* the conductance always has the maximum at the same *u*_0_ = *u*_*m*_ ≈ 3.5. Inset: *g* versus *v* for the channel with resonant potential *u*_0_ = *u*_*m*_.

Furthermore, we demonstrate in Figure 7(a) the current-versus-voltage curves, *I*(*v*) (*I*-*V*) characteristics, extracted from Figure 5 for several channels of different *u*_0_. The highest curve describes the *I*-*V* characteristics of the resonantly self-optimized channel with *u*_0_ = *u*_*m*_. It is clear that all the *I*(*v*)-curves presented in this figure are nonlinear, indicating the nonohmic property of the channel model studied. In this case, the channel ion conductance, defined as the ratio of *I* to *v* [30], becomes dependent on the applied voltage. The *I*(*v*)-curves in Figure 7(a) reveal that as *v* increases the conductance *g* = *I*/*v* decreases fast first at small *v*, reaches a minimum at *v* ≈ 2.5–3, and then slightly increases at higher *v* (see, e.g., the inset in Figure 7(b) for the case *u*_0_ = *u*_*m*_). The voltage, where the conductance gets minimal, depends on the potential *u*_0_ and the reservoir particle concentrations. To look for a possible relation between the channel ion conductance and the resonant channel potential *u*_*m*_ associated with the current (Figure 6), we present in Figure 7(b) the conductance *g* calculated for channels of different potentials *u*_0_ at the same voltage *v*. Remarkably, at any *v* the conductance *g* always has the maximum at the same *u*_0_ = *u*_*m*_ as that identified in Figure 6 for the current. So, Figure 7(b) gives one more demonstration for the resonantly self-organized property of channels in facilitating the transmembrane particle movement. The *v*-dependence of *g* in this figure is related to the sublinear behavior of the *I*-*V* curves in Figure 7(a) as just discussed above (see the inset in Figure 7(b)). Note that such the sublinearity of calculated *I*-*V* curves qualitatively resembles experimental data reported [22, 23].

**Figure 8:**
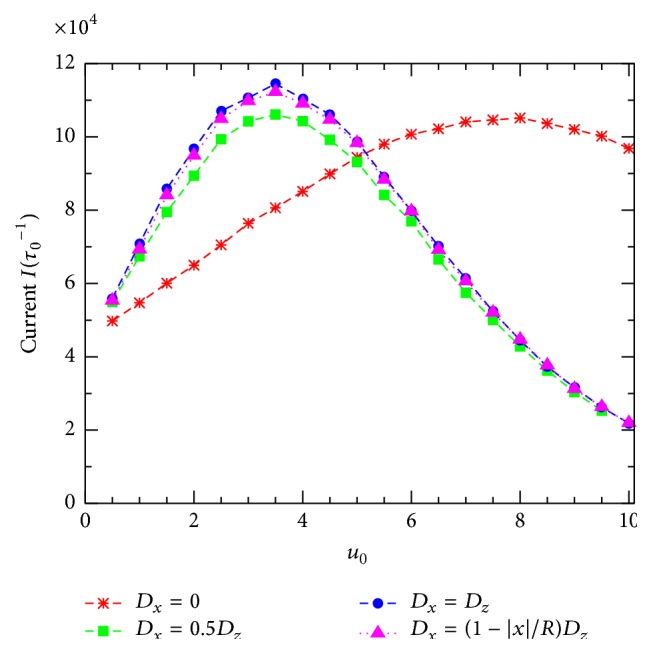
The *I*(*u*_0_) curves for different *D*_*x*_ (indicated in the figure) are compared to show the role of the transverse diffusion in the 2D diffusion model considered (*v* = 5, *D*_*z*_ = 0.5*D*_0_; other parameters are the same as in Figure 2).

Finally, Figure 8 compares the *I*(*u*_0_)-curves obtained for different *D*_*x*_ in showing the role of the transverse diffusion in the 2D diffusion model under study. Four cases presented are (1) *D*_*x*_ = 0, implying the 1D diffusion, (2) *D*_*x*_ = 0.5*D*_*z*_, implying an anisotropically 2D diffusion with *D*_*x*_ constant and smaller than *D*_*z*_, (3) *D*_*x*_ = *D*_*z*_, implying an isotropically 2D diffusion, and (4) *D*_*x*_ = [1 − (|*x*|/*R*)]*D*_*z*_ used in this work (see corresponding symbols given in the figure). Obviously, the curve in the case of 1D diffusion is largely separated from the rest, showing an essential role of the transverse diffusion. In this limiting case there is also the maximum in the *I*(*u*_0_)-curve; however, the current peak is lower and the resonant potential is much larger (≈8), compared to those for 2D diffusion models. Interestingly, all the three 2D diffusion *I*(*u*_0_) curves with *D*_*x*_ different on the behavior or the value show very similar forms with the same resonant potential *u*_*m*_ = 3.5. In addition, the isotropic 2D diffusion model, *D*_*x*_ = *D*_*z*_, provides the highest current peak.

## 4. Conclusions

We have considered an anisotropic 2D diffusion of a charged molecule (particle) through a large biological channel under an external voltage. Connecting the two reservoirs with different particle concentrations, the channel is modeled as a rigid cylinder characterized by the three structure parameters: the radius, the length, and the surface density of the negative charges of channel interior-lining. These negative charges induce inside the channel a potential that is uniquely determined by the channel structure parameters and that critically affects the transmembrane particle movement. The suggested model is rather phenomenological so that the channel-induced potential can be calculated exactly. Nevertheless, it serves well to gain an understanding of the physical mechanism of the channel-facilitated particle movement. More detailed quantitative models are required to describe concrete realistic biological channels (see, e.g., [31, 32]).

Our study is concentrated on showing the influences of this channel-induced potential and the external voltage on the typical dynamical characteristics of particles such as the translocation probabilities, the average translocation times, the net current, and the channel ion conductance. It was shown that while the external voltage does not cause any especial effect, the channel potential increases both the translocation probabilities and the average translocation times. And, surprisingly, studies demonstrated a single average translocation time that is equally applied for the particles passing the channel in two contrary directions, regardless of even the directed influence of the external voltage.

The most interesting result appeared in examining the particle current. It was shown that at a given temperature the channel with appropriate structure parameters can induce the resonant potential that effectively facilitates the transmembrane movement of the particles of a given valence, resulting in a very large net particle current across the channel. In other words, to facilitate the transmembrane particle movement the channel should be naturally self-optimized so that its potential coincides with the resonant one. The resonant potential is an intrinsic characteristic of the channel and facilitating the transmembrane particle movement is an intrinsic property of biological channels, independent of the external factors such as the external voltage or the particle concentration gradient. In addition, the observed CFPM is very selective in the sense that a channel of definite structure parameters can facilitate the transmembrane movement of only particles of proper valence at corresponding temperatures. Calculated current-voltage characteristics also show that the channel model is nonohmic. The full characteristics of conductance exhibit an absolute maximum at the same resonant channel potential as that identified in the currents.

It should be conclusively noted that all the results presented above are principally related to the considered single particle model, neglecting all the effects associated with the many-particle couplings, the particle size, and the potential induced by particle itself. So, these results might serve as an argument for further studies.
